# New records of parasitic copepod (Crustacea, Siphonostomatoida, Pennellidae) found on the body surface of two cetacean species in China

**DOI:** 10.3897/BDJ.11.e98914

**Published:** 2023-02-20

**Authors:** Qianhui Zeng, Yejie Lin

**Affiliations:** 1 Laboratory of Marine Biology and Ecology, Third Institute of Oceanography, Ministry of Natural Resources, Xiamen, China Laboratory of Marine Biology and Ecology, Third Institute of Oceanography, Ministry of Natural Resources Xiamen China; 2 Hebei Key Laboratory of Animal Diversity, College of Life Science, Langfang Normal University, Langfang, China Hebei Key Laboratory of Animal Diversity, College of Life Science, Langfang Normal University Langfang China

**Keywords:** new distribution, taxonomy, cetacean external parasites, *Pennella*

## Abstract

**Background:**

*Pennellabalaenoptera* Koren & Danielssen, 1877 (Crustacea, Siphonostomatoida, Pennellidae) is so far known as the largest copepod species and has only been found as a parasite on marine mammals. Previous studies on parasitic copepods in China only focused on those found on economic fishes, no records of *P.balaenoptera* having ever been reported before.

**New information:**

A Bryde's whale (*Balaenopteraedeni* Anderson, 1879) and a pygmy sperm whale [*Kogiabreviceps* (Blainville, 1838)] were respectively stranded on the eastern and southern coast of China in 2021 and 2022. A total of eight parasitic copepods found on their body surface were sampled and examined. The parasites were identified as *Pennellabalaenoptera*, based on morphological characteristics and measurements. Descriptions and photomicrographs of this newly-recorded species from China are given in the present study.

## Introduction

Species of the genus *Pennella* Oken, 1815 (Crustacea, Siphonostomatoida, Pennellidae) are medium-sized parasitic arthropods that predominantly parasitise on the surface of marine fishes and marine mammals (Hogans 2017). Most of them are highly host-specific. Amongst them, *Pennellabalaenoptera* Koren & Danielssen, 1877 is known as the largest species and has only been found on marine mammals, including cetaceans (both baleen whales and toothed whales) (e.g. Ciçek et al. (2007); Danyer et al. (2014)) and pinnipeds (Dailey et al. 2002). *P.balaenoptera* is sexually dimorphic, the male is known as free-swimming and does not attach to hosts (Turner 1905), while female needs to attach to hosts for reproduction (Danyer et al. 2014). The entire life histories of both sexes are not yet well understood.

Cetaceans are large-sized, fast-moving and wide-ranging animals that live in the water throughout their lives; thus, their external parasite samples are not easily collected in the wild. Most cetacean external parasites were collected from their stranded carcasses, including parasitic copepods (e.g. Aznar et al. (2005)). Previous research on parasitic copepods in China only focused on those parasitic on economic fishes. This paper reports the first records of *P.balaenoptera* found on the cetaceans stranded in the eastern and southern coast of China, enriching the distribution data on parasitic copepods.

## Materials and methods

**Figure 1.**
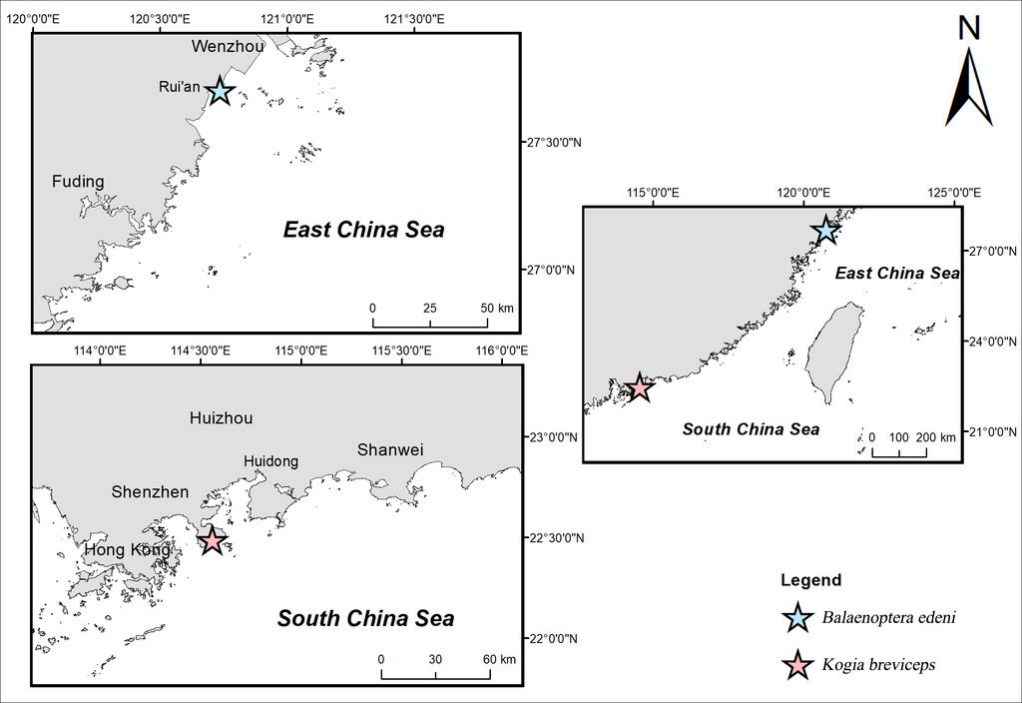
Distribution records of *Pennellabalaenoptera* in China.

**Figure 2.**
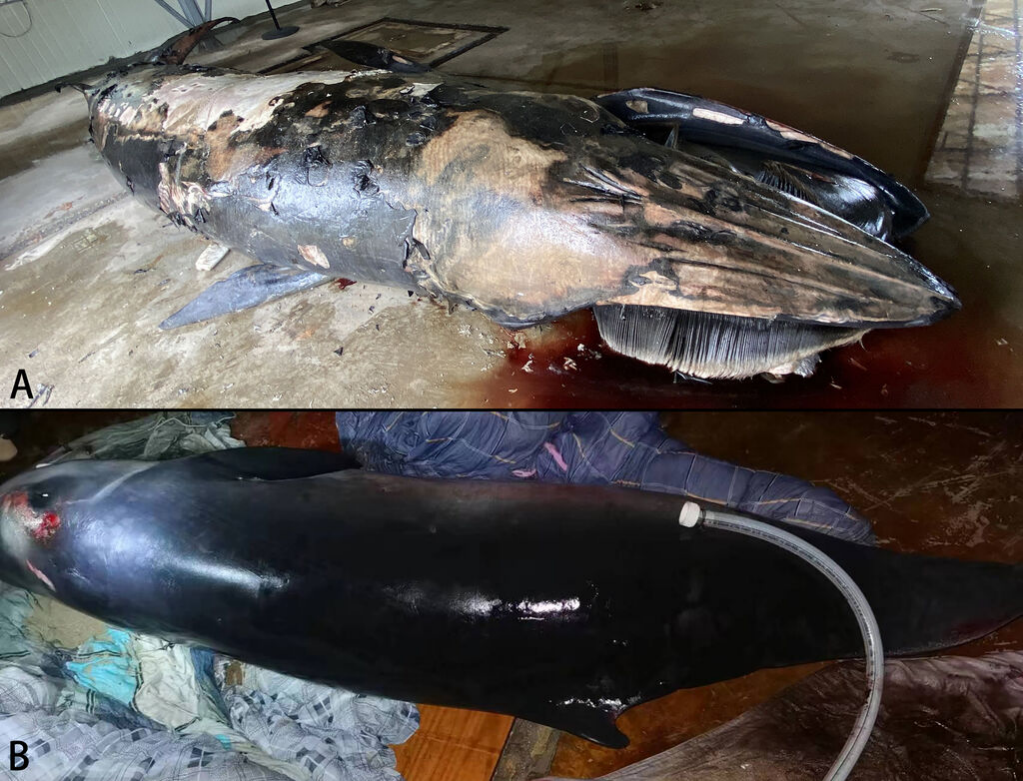
Hosts of *Pennellabalaenoptera*; **A.** *Balaenopteraedeni*; **B.** *Kogiabreviceps*.

On 27 July 2021, a 6.85 m long baleen whale was found stranded on a mudflat in Rui'an, Wenzhou (Fig. 1) and died later. It was identified as *Balaenopteraedeni* Anderson, 1879 (Bryde's whale). based on morphology (Fig. 2A). The whale carcass was preserved in a frozen environment (about -20°C) until 31 July 2021, when it was dissected for necropsy. The whale's blubber was thin. Many healed bite marks caused by *Isistius* sp. (cookiecutter sharks) were found on its ventral side, with the densest, 13 bite marks on a 20 cm × 20 cm skin area. In addition, numerous traces of parasitic infestation were found on the trunk surface. Most of the parasites were incomplete and, therefore, only six parasite specimens were taken from this host to study.

On 21 May 2022, a 2.52 m long toothed whale was found stranded on a sandy beach in Xichong, Shenzhen (Fig. 1) and died after several failed rescues. It was identified as *Kogiabreviceps* (Blainville, 1838) (pygmy sperm whale), based on morphology (Fig. 2B). Two brush-like structures were found near the dorsal fin base of the animal, with connecting parts buried under the skin, which were, thus, suspected to be parts of parasites. After the animal died, the two potential parasites were carefully pulled out and preserved in 75% ethanol. The whale carcass remained to be dissected for necropsy, while the two parasite specimens were examined and described.

The specimens were all preserved in 75% ethanol. They were later soaked in trypsin solution for 12 hours to dissolve and wash off the residual host cetacean tissues on their body surface. The processed specimens were examined under a LEICA M205C body view microscope. Images were captured with an Olympus C7070 zoom digital camera (7.1 megapixels) and superimposed by Helicon Focus 6.7.1 and then processed in Adobe Photoshop CC 2018. All measurements are in millimetres (mm) and were obtained with an Olympus SZX16 stereomicroscope with a Zongyuan CCD industrial camera.

The specimens are currently deposited in the Institute of Zoology, Chinese Academy of Sciences in Beijing (IZCAS). The terminology used in the text and figures follows Abaunza et al. (2001) and Hogans (2017). Abbreviations used in the text and figures: AR, antennary region; FA, first antenna; O, oviduct orices; R, rami; SA, second antenna; SL, swimming legs.

Based on morphological features, the specimens are identified as *Pennellabalaenoptera*. Since male *P.balaenoptera* is free-living like other non-parasitic copepods, while the female is parasitic, the samples obtained in this study are all females.

## Taxon treatments

### Pennella balaenoptera

Koren & Danielssen, 1877

26F6B373-3FC2-5610-89E6-515C32B52CA9

https://www.catalogueoflife.org/data/taxon/76JPB

#### Materials

- **Type status:** Other material. **Occurrence:** sex: 6 females; occurrenceID: DAA4F37B-61EF-5926-AAF9-1FDF6957D477; **Taxon:** scientificName: *Pennellabalaenoptera*; nameAccordingTo: Koren, J. & D.C. Danielssen. (1877). En ny Art af Slaegten *Pennella*. (A new species of the genus *Pennella*). Fauna Littoralis Norvegiae. 3:157–163, pl.16, figs.1–9.; kingdom: Animalia; phylum: Arthropoda; class: Copepoda; order: Siphonostomatoida; family: Pennellidae; genus: Pennella; taxonRank: species; taxonomicStatus: accepted; **Location:** continent: Asia; country: China; stateProvince: Zhejiang; county: Wenzhou, Rui'an; locality: Mudflat; verbatimLatitude: 27.7099°N; verbatimLongitude: 120.7231°E; **Event:** year: 2021; month: 7; day: 27; verbatimEventDate: 27-07-2021; eventRemarks: the material is found parasitic on a stranded Bryde's whale (*Balaenopteraedeni*); **Record Level:** institutionID: IZCAS-Ar43739–Ar43744
- **Type status:** Other material. **Occurrence:** sex: 2 females; occurrenceID: 9AD18284-D7A6-5CDC-A2BA-CFE578576A1A; **Taxon:** scientificName: *Pennellabalaenoptera*; nameAccordingTo: Koren, J. & D.C. Danielssen. (1877). En ny Art af Slaegten *Pennella*. (A new species of the genus *Pennella*). Fauna Littoralis Norvegiae. 3:157–163, pl.16, figs.1–9.; kingdom: Animalia; phylum: Arthropoda; class: Copepoda; order: Siphonostomatoida; family: Pennellidae; genus: Pennella; taxonRank: species; taxonomicStatus: accepted; **Location:** continent: Asia; country: China; stateProvince: Guangdong; county: Shenzhen, Xichong; locality: Sandy beach; verbatimLatitude: 22.4875°N; verbatimLongitude: 114.5568°E; **Event:** year: 2022; month: 5; day: 21; verbatimEventDate: 21-05-2022; eventRemarks: the material is found parasitic on a stranded pygmy sperm whale (*Kogiabreviceps*); **Record Level:** institutionID: IZCAS-Ar43745–Ar43746

#### Description

**Figure 3.**
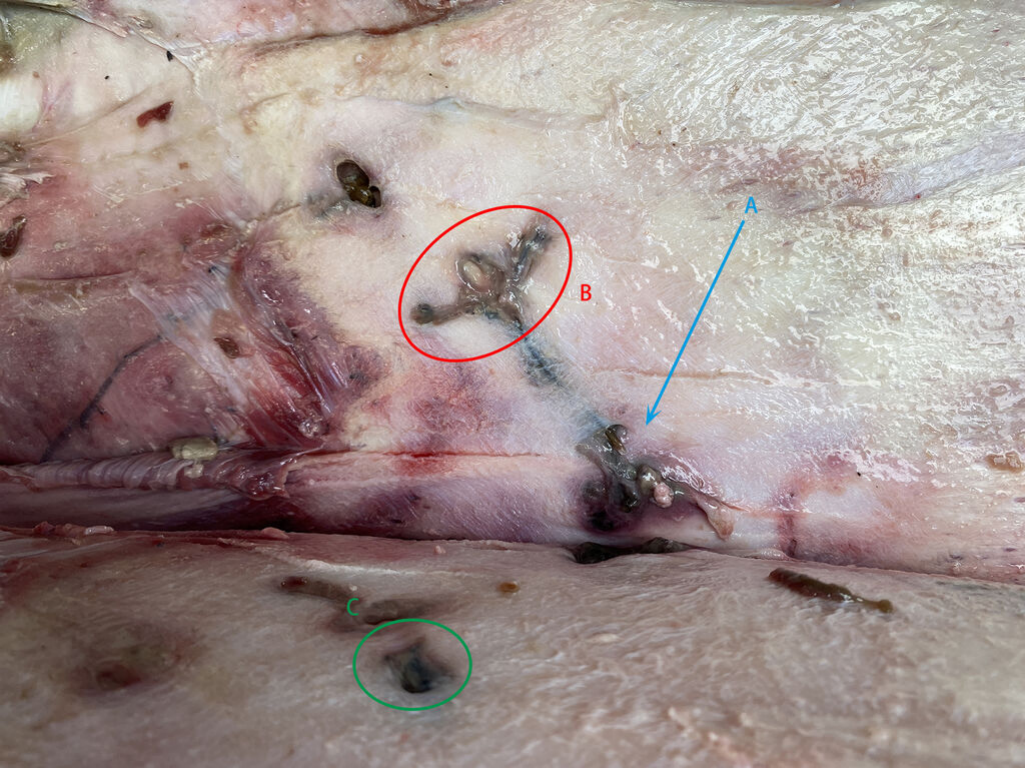
*Pennellabalaenoptera*; **A.** Body; **B.** Head anchorage area; **C.** Body through epidermal trace.

These parasites were rooted in the host bodies by elongated anchor-like structures, which passed through the blubber and the skin (Fig. 3A, B) with their ends exposed, leaving small round holes on the body surface (Fig. 3C).

**Figure 4.**
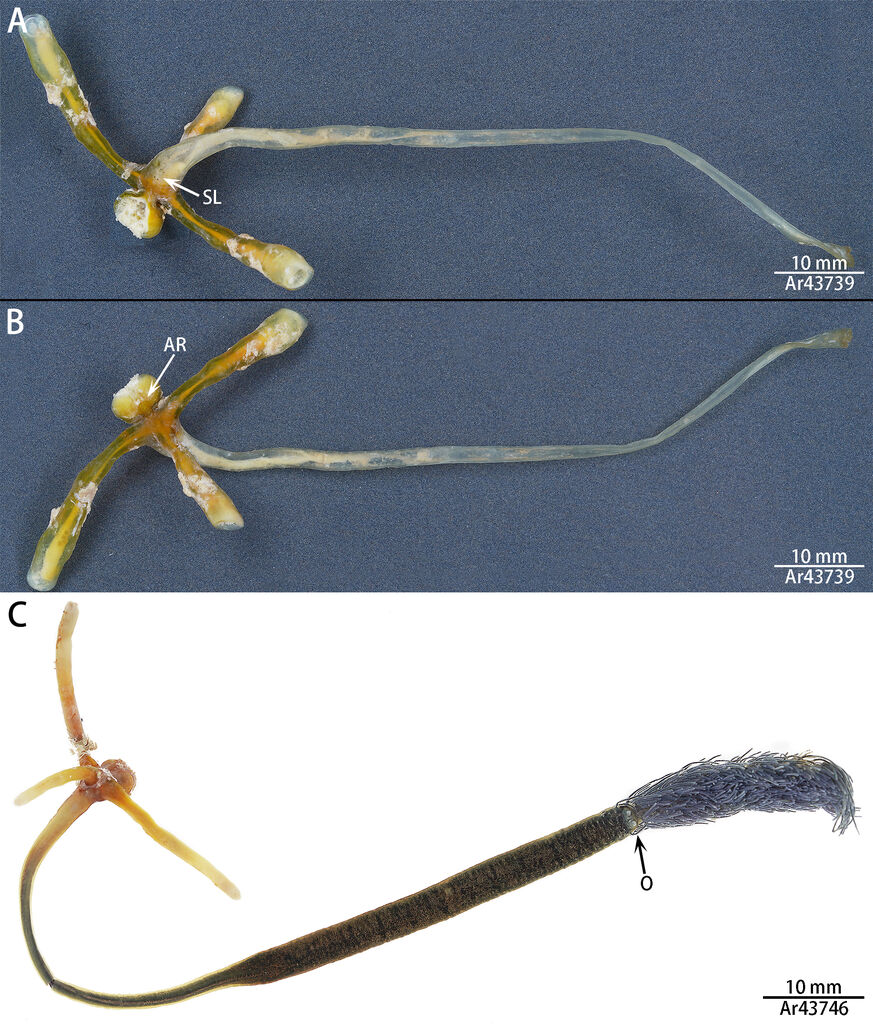
Habitus of *Pennellabalaenoptera*; **A.** Dorsal view; **B, C.** Ventral view.

One specimen (IZCAS-Ar43746) (Fig. 4C):

"Head": 3.64 long, 4.12 wide; Holdfast horns: left 11.32 long, 2.02 wide; right 15.23 long, 1.62 wide; dorsal 11.53 long, 1.51 wide; "Neck": 43.61 long, 1.47 wide; Trunk: 40.53 long, 3.64 wide; Abdomen: 29.64 long.

Colouration. Specimens cephalothorax yellow; neck, trunk and abdomen dark brown in ethanol. Divided into three regions: cephalothorax, carapace and abdomen. Ovisacs missing.

**Figure 5.**
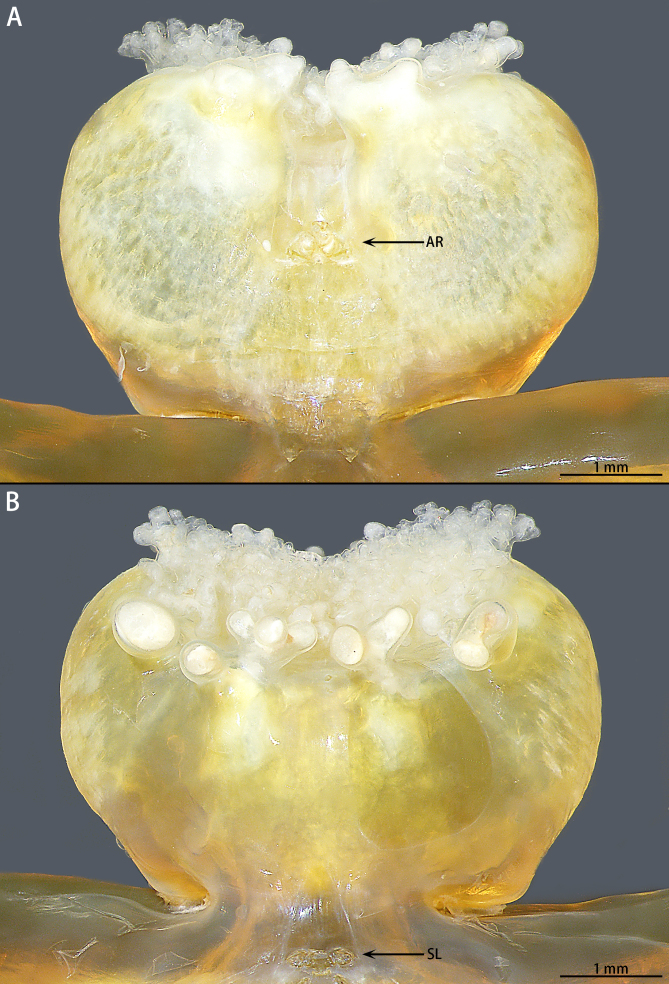
Cephalic region of *Pennellabalaenoptera*; **A.** Ventral view; **B.** Dorsal view.

**Figure 6.**
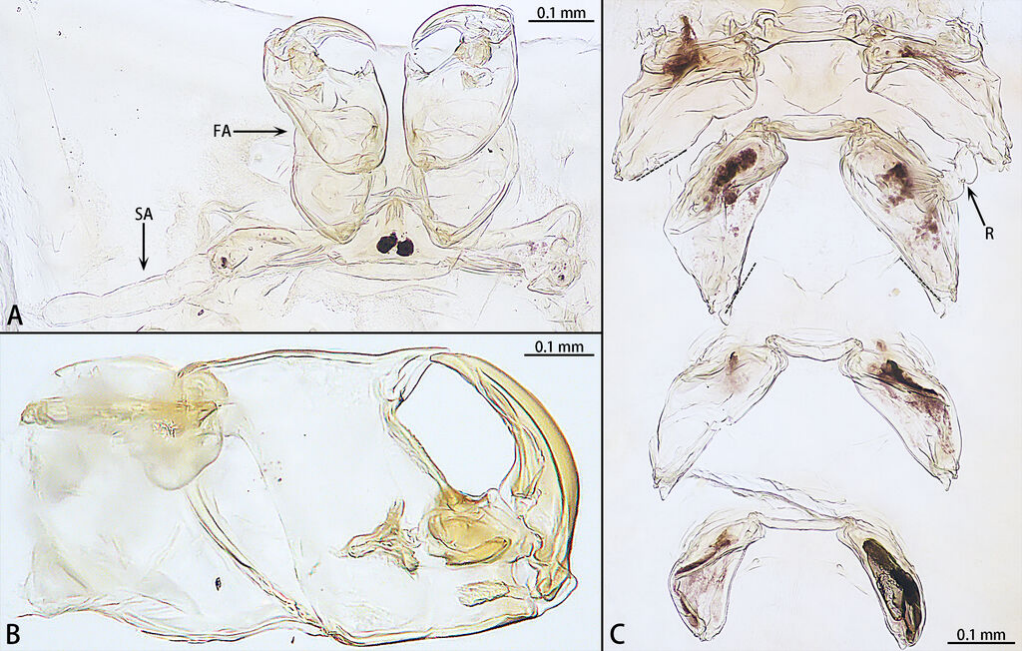
Antennary region and swimming legs region of *Pennellabalaenoptera*; **A.** Antennary region; **B.** Second antenna of *Pennellabalaenoptera*, dorsal view; **C.** swimming legs region.

Cephalothorax. Divided into cephalic and thoracic regions, separated by a constricted region between the two parts. Cephalic region spherical, slightly wider than long, abdominal side to the front mask mastoid, with dense, different sized spherical mastoid branches (Fig. 5), the anterior side of the mastoid area has a mouth centrally; the abdominal surface has a slender depression in the middle with two pairs of antennae inside the depression, which are extremely small (Fig. 5A, Fig. 6A); the first pair of antennae three segmented, whisker-like, different in length, terminal with conspicuous setae, second pair of antennae two-segmented, with pincers at the end (Fig. 6B). The anterior part of the dorsal thoracic region with four pairs of reduced swimming legs, paddle-shaped. The first two pairs closely spaced, terminal with rami, the rami of first pair of swimming legs two-segmented, with conspicuous setae, the rami come off the second pair of swimming legs; the last two pairs are unbranched and widely spaced, about five times the spacing between the first two pairs of legs (Fig. 6C). Three holdfast horns, digitiform.

**Figure 7.**
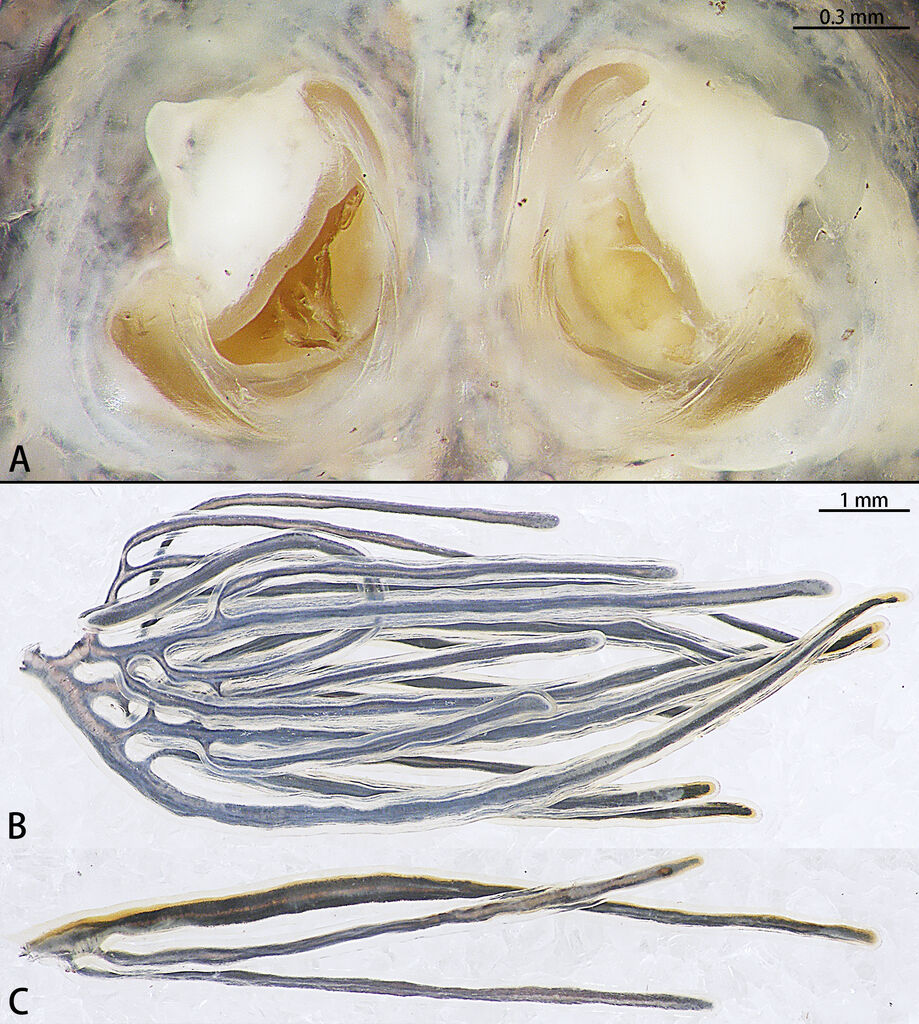
Oviduct orices (**A**) and abdominal plumes (**B, C**) of *Pennellabalaenoptera*.

Thoracic region. The neck, the longest part of this region extremely elongated with a wider trunk and accounts for 72% of the body, transversely striated, the orifices of the oviducts appear at the posterior end of the trunk, crescent-shaped (Fig. 7A).

Abdomen. Cylindrical, with lateral, feather-like processes. Each of these feather-like processes has a different shape (Fig. 7B, C).

Ovisacs missing.

#### Biology

Hosts: *Balaenopteraedeni*, 6.85 m long; *Kogiabreviceps*, 2.52 m long.

## Discussion

The species *Pennellabalaenoptera* was first described by Koren and Danielssen (1877), based on specimens collected from *Balaenoptera* spp. from the Faroe Islands. It is currently the largest copepod and the only *Pennella* species parasitic on marine cetaceans (Fraija-Fernández et al. 2018). *P.balaenoptera* parasitism has also been reported from other marine mammals, such as northern elephant seals [*Miroungaangustirostris* (Gill, 1866)] (Dailey et al. 2002). Interspecific morphological variation in female *P.balaenoptera* is high, with the most dramatic variation presented in the neck and fixation angle. Consequently, synonymy with this species is more common (Hogans 2017). Female of this species can be distingulished by the terminal segment of the second antenna and the only one ramus is at the terminus of the first two pairs of swimming legs.

As a sexually dimorphic species, female *P.Balaenoptera* is large and half-buried in the host's body, appearing as a hanging tag or filament (Vecchione and Aznar 2014). It can be discovered easily, unless its body is broken, especially when the host is dead. Unlike some pennellids that only parasitise on the fins of hosts (Venmathi-Maran et al. 2012), female *P.balaenoptera* generally burrows on the host's dorsal and ventral trunk (Raga et al. 2009), but sometimes does not show regular patterns. They can also parasitise on the head, flanks or tail stock of hosts (Table 1). Previous research indicated that they tend to parasitise on regions with dense vein vessels under the skin (e.g. Danyer et al. (2014)), which coincides with the description that they feed on the host's blood and body fluids (Dailey 2001, Aznar et al. 2005). Whether the infested region has variation amongst host species remains to be further studied.

Severe infestations caused by *P.balaenoptera* are often found on weakened hosts (Aznar et al. 2005, Vecchione and Aznar 2014), which is also indicated in the present study. In the first case, the host had thin blubber and, besides the numerous *Pennella* infestations, lots of cookiecutter shark bites were distributed on its skin, revealing its bad health condition and slow-moving speed.

Compared to records of *P.balaenoptera* found on the rorquals (family Balaenopteridae), records found on the family Kogiidae are scarce. It was firstly reported attached to a pygmy sperm whale (*Kogiabreviceps*) in 1951 (Hubbs 1951). The present study is maybe the second record of this parasite on the pygmy sperm whale.

Previous studies on *P.balaenoptera* morphology, life history and parasitism effects were mainly concentrated in the Mediterranean district (Table 1). In East Asia, prevalence of *P.balaenoptera* on common minke whales (*B.acutorostrata*) from the western north Pacific has been reported (Uchida et al. 1998, Uchida and Araki 2000), but no records have ever been reported from China. This paper presents the first *P.balaenoptera* record from China with a series of photomicrography pictures and enriches the parasitic copepod distribution database.

## Supplementary Material

XML Treatment for Pennella balaenoptera

## Figures and Tables

**Table 1.** Table 1: Cetacean hosts and microhabitats in some *Pennellabalaenopterae* infestation cases.

Host	Host family	Microhabitat	Location	Reference
*Balaenopteraacutorostrata* (Common minke whale)	Balaenopteridae	All but head region	Around Iceland	Ólafsdóttir and Shinn (2013)
*B.bonaerensis* (Antarctic minke whale)	Balaenopteridae	Flanks	Southern Ocean	Ten et al. (2022)
*B.physalus* (Fin whale)	Balaenopteridae	Flanks (Sampled regions); Abdomen	North-eastern Mediterranean coast of Turkey; Mediterranean Sea	Ciçek et al. (2007), Notarbartolo-Di-Sciara et al. (2003)
*B.edeni* (Bryde's whale)	Balaenopteridae	Dorsal region, flanks and ventral side	Eastern coast of China	Present study
*Grampusgriseus* (Risso's dolphin)	Delphinidae	Head region (melon) and behind the mammary gland	North-eastern Mediterranean coast of Italy	Cornaglia et al. (2010)
*Stenellacoeruleoalba* (Striped dolphin)	Delphinidae	Flanks	Mediterranean coast of Spain	Aznar et al. (1994)
*Phocoenaphocoena* (Harbour porpoise)	Phocoenidae	Flanks and caudal peduncle	Aegean coast of Turkey	Danyer et al. (2014)
*Kogiabreviceps* (Pygmy sperm whale)	Kogiidae	Head	Eastern Pacific coast of USA	Hubbs (1951)
*K.breviceps* (Pygmy sperm whale)	Kogiidae	Dorsal region (near the dorsal fin)	Southern coast of China	Present study
